# Childhood traumatic experiences and addiction-like eating behaviors: the mediating roles of attachment, mentalization, and emotional eating

**DOI:** 10.1186/s40337-025-01473-x

**Published:** 2025-12-23

**Authors:** Alessandro Alberto Rossi, Stefania Mannarini

**Affiliations:** 1 Department of Philosophy, Sociology, Education, and Applied Psychology, Section of Applied Psychology, University of Padova, Padova, Italy; 2 Center for Intervention and Research on Family Studies-CIRF-Department of Philosophy, Sociology, Education, and Applied Psychology, Section of Applied Psychology, University of Padova, Padova, Italy

**Keywords:** Food addiction, Addiction-like eating behaviors, Childhood traumatic experiences, Attachment anxiety, Mentalization, Low diet control, Eating addiction

## Abstract

**Introduction:**

Childhood traumatic experiences (CTEs) represent a significant vulnerability factor for addiction-like eating behaviors, yet the underlying developmental pathways remain poorly understood. According to infancy research and attachment theory, this study tested a comprehensive mediation model examining how CTEs contribute to addiction-like eating patterns (i.e., appetite drive and low diet control) through attachment insecurity, impaired reflective functioning, and emotional eating.

**Method:**

A cross-sectional study was conducted on a conventional non-clinical sample 1,014 Italian adults. Participants completed a set of validated and standardized scales. Structural equation modeling with latent variables and bootstrap resampling (10,000 iterations) was employed to test the hypothesized model.

**Results:**

The model demonstrated adequate fit and supported all hypotheses. CTEs significantly predicted both attachment anxiety, attachment avoidance, and impaired reflective functioning. Attachment anxiety, but not avoidance, mediated the relationship between CTEs and impaired reflective functioning. Also, impaired reflective functioning significantly predicted emotional eating, which in turn predicted both appetite drive and low dietary control. The complete mediation pathway was statistically significant, explaining 53.5% of variance in appetite drive and 20.6% in dietary control.

**Discussion:**

These findings provide the first empirical support for a trauma-based developmental model of addiction-like eating behaviors. The results highlight the central role of attachment anxiety and impaired reflective functioning in linking early relational trauma to emotional eating and food addiction-like patterns. Clinical implications suggest that mentalization-based interventions targeting attachment insecurity and emotion regulation may be particularly beneficial for individuals with trauma histories and problematic eating behaviors.

## Abstract (plain language summary)

People who experienced difficult or harmful relationships with caregivers during childhood, such as abuse or neglect, are more likely to develop unhealthy eating patterns that resemble addiction. This study explored how this happens. We surveyed over 1,000 Italian adults and found that childhood trauma creates a chain reaction. First, these early negative experiences often lead people to feel anxious in their relationships and struggle to understand their own emotions and those of others. When people cannot recognize or manage their feelings well, they may turn to food for comfort, eating to cope with stress rather than hunger. Over time, this emotional eating can develop into addiction-like behaviors, including intense cravings for certain foods and difficulty controlling how much they eat. Our findings suggest that treating problematic eating patterns requires addressing more than just food and diet. Therapies that help people better understand their emotions, improve their relationships, and develop healthier ways to manage stress may be especially helpful for those who experienced childhood trauma. Understanding these connections can lead to more effective support for people struggling with food-related difficulties.

## Introduction

Disordered eating behaviors related to excessive food intake and overeating include a range of maladaptive patterns triggered by dysfunctional responses to food-related stimuli [1]. These behaviors have garnered increasing clinical interest because of their strong links with overweight and obesity [2], which are chronic conditions shaped by complex medical, environmental, and psychological influences [3]. Studies have described different forms of excessive food consumption and overeating, from compulsive grazing to binge eating episodes [4–6]. These patterns often share transdiagnostic features [7] and symptoms suggestive of food-related addictive processes, which may underlie and perpetuate maladaptive (over)eating behaviors [8].

These disordered eating behaviors are frequently associated with the repeated overconsumption (i.e., consuming food in quantities beyond physiological needs or intended amounts) of ultra-processed foods (UPFs). These foods are highly palatable and can trigger addiction-like eating responses and symptoms in certain individuals—particularly those with vulnerabilities related to reward sensitivity, emotion dysregulation, or early adverse experiences—which is commonly referred to as food addiction (FA) [9–12]. UPFs rich in sugar, salt, and fat [13–15] have been shown to contribute to the onset and persistence of overweight and obesity, partly because of their addictive properties [16–18] and their capacity to engage neural reward systems [19–21] and activate brain circuits [22] involved in addictive behavior and substance-related and addictive disorders (SRADs).

Thus, FA not only overlaps with transdiagnostic core features of disordered eating behaviors and eating disorders (EDs) but also exhibits traits common in SRADs, creating a self-reinforcing and cyclical pattern [23, 24].

Indeed, individuals with FA develop addiction-like symptoms that are strongly polarized toward food. They experience tolerance, whereby increasing quantities of food—particularly UPFs and palatable ones—are needed to achieve the same level of satisfaction [25] or emotional relief [4, 23]. Additionally, individuals with FA may experience withdrawal-like symptoms, such as irritability, restlessness, and intense food cravings [25, 26]. These symptoms can trigger intrusive thoughts and compulsive eating behaviors (i.e., emotional eating) [8] aimed at restoring the pleasurable sensations associated with food consumption [4, 5], often resulting in uncontrolled eating episodes [8, 27], such as grazing or binge eating. Indeed, core features of FA include heightened appetite drive—characterized by increased responsiveness to food-related cues and tendencies toward overeating—as well as reduced dietary control, marked by difficulty regulating food intake and adhering to structured eating patterns [4, 5, 27, 28]. These manifestations reflect the persistent cognitive and behavioral dysregulation around food [8] that characterizes FA. These manifestations are not limited to binge eating but span a spectrum of maladaptive eating behaviors [23, 24] that compromise self-regulation [27, 29, 30]. Furthermore, FA symptoms are frequently observed across different ED diagnoses [23, 31, 32], suggesting that FA may operate as a transdiagnostic construct [33–36]. This finding reinforces the complex and dual clinical nature of FA: on the one hand, it involves neurobiological and behavioral features akin to those of SRADs [22]; on the other hand, it encompasses the psychological and emotional processes commonly associated with EDs [8, 27].

Consequently, given the dual nature of FA, it is plausible that FA may share etiological pathways with both SRADs and EDs [27, 37]. Although various risk factors and triggering cues have been identified in the literature [38, 39], their specific roles and the way they interact are still not entirely clear. Moreover, among the factors implicated in both conditions, childhood traumatic experiences (CTEs)—including emotional, physical, and sexual abuse, as well as emotional and physical neglect occurring within the caregiving context [40]—have emerged as a significant and shared vulnerability [41–48].

Within the theoretical framework of infancy research and attachment theory, CTEs (particularly emotional maltreatment) have been consistently linked to disordered eating behaviors [27, 48–52]. The literature indicates that such early traumatic adversities are consistently associated with increased vulnerability to dysfunctional eating patterns [27, 49, 53], as well as to FA [27, 54, 55]. Although the body of research is still developing, current findings point to a relationship between CTEs, especially emotional maltreatment, and the emergence of FA symptoms [56–61] and addiction-like eating behaviors [27], with more severe trauma often linked to greater symptom intensity [62].

Consistent with attachment theory, CTEs are known to undermine the development of secure attachment patterns [49, 63, 64], often leading to relational insecurities (e.g., attachment anxiety and attachment avoidance) and increased vulnerability to psychopathologies, such as SRADs and EDs [44, 49, 65]. According to attachment theory, CTEs can interfere with the development of the attachment system by undermining the caregiver’s role as a secure base and disrupting the formation of secure attachment bonds [42, 66], potentially leading to difficulties in self-regulation and a fragmented self-concept [30, 67]. These outcomes are often associated with emotionally unresponsive or misattuned caregiving, which impairs the child’s ability to use the caregiver as a secure base—a core component of attachment security [30, 68]. Traumatic interactions with caregivers during childhood shape internal working models—mental representations of the self, others, and interpersonal relationships—that influence emotional regulation, self-perception, and behavior across the lifespan, including into adulthood [49, 69]. In adulthood, attachment is typically conceptualized along two dimensions: anxiety and avoidance [70, 71]. Attachment anxiety reflects a negative view of the self and a heightened need for closeness, often accompanied by a fear of rejection. In contrast, attachment avoidance involves a positive self-view paired with mistrust of others and discomfort with intimacy [70, 72]. Both forms of attachment insecurity have been implicated in disordered eating behaviors, EDs, and SRADs, particularly through their impact on emotion regulation strategies [73, 74]. Moreover, although findings show some variability in the relative contributions of attachment anxiety and avoidance, attachment anxiety is generally considered a stronger risk factor for the development of both substance use behaviors and dysfunctional eating patterns related to overeating [74] and, thus, likely addiction-like eating behaviors. Importantly, the literature suggests that attachment insecurities may mediate the relationship between CTEs and eating behaviors [49, 63, 64]. In the presence of attachment insecurity, higher rates of early abuse and neglect have been observed among individuals with disordered eating behaviors [64] and in nonclinical samples [49], supporting a mediating role for attachment. Specifically, a study by Musetti and colleagues (2023) revealed that patterns of attachment insecurity mediate the association between CTEs and disordered eating behaviors via mentalization (i.e., impaired reflective functioning); however, only attachment anxiety significantly contributes [49].

Consistent with these findings, the literature shows that attachment and mentalization are deeply interconnected [75]. In fact, relational disruptions can impair the development of fundamental interpersonal functions, such as reflective functioning [42, 76, 77].

According to Fonagy, the construct of mentalization, often referred to as reflective functioning, is a capacity rooted in early child–caregiver interactions [78, 79]. Reflective functioning refers to the ability to understand and interpret one’s own and others’ behaviors by linking them to underlying mental states [80] such as thoughts, emotions, and intentions [75, 81]. This function allows individuals to attribute meaning to actions and experiences without relying solely on external cues or validation [80, 81]. For instance, adequate reflective functioning enables individuals to recognize and understand the emotional origins of their eating behaviors, such as identifying stress as the driver of an eating urge rather than physiological hunger. In contrast, impaired reflective functioning (hypomentalization) results in automatic, impulse-driven eating without awareness of the underlying emotional triggers.

Indeed, when reflective functioning is impaired, such as in the presence of CTEs or attachment insecurities, this crucial interpersonal ability becomes compromised [81]. Individuals with impaired reflective functioning (i.e., hypomentalization) operate automatically and unconsciously, hindering their capacity to interpret and regulate behavior [82], especially in emotionally intense situations [83]. Such impairment leads to a rigid and distorted perception of mental states, causing individuals to experience reality in a fragmented, prementalized way [42, 84], where internal emotions feel overwhelming and disorganizing [49, 81, 85, 86]. Consequently, to defend against these distressing thoughts and emotions [82, 87, 88], individuals may rely on maladaptive coping mechanisms [89, 90] or external regulators [84, 86, 91], such as substance use or food (i.e., UPFs), that provide an immediate response and relief [68, 76]. Research highlights hypomentalization as a key characteristic among those with disordered eating behaviors [27, 29, 49, 84]. Consequently, when faced with aversive internal states such as anxiety or intrusive thoughts, individuals with low reflective functioning often engage in impulsive, emotionally driven eating behaviors [81, 89, 90, 92], commonly known as emotional eating [93, 94].

As psychological stress or arousal intensifies, particularly following traumatic or highly stressful events, milder coping strategies such as emotional eating often become insufficient [8, 27, 91]. Individuals may then resort to more severe, maladaptive behaviors, including compulsive, repetitive, or binge eating, as attempts to suppress or numb overwhelming negative emotions [8, 27, 42, 88, 95, 96]. This escalation typically leads to diminished control over eating and diet and fosters addiction-like eating patterns [27].

In summary, individuals exposed to CTEs may develop insecure attachment styles linked to impairments in reflective functioning (hypomentalization) [49, 81, 85]. These deficits in self-regulation [29, 30] require an external object to restore internal emotional regulation, such as food [49, 84, 86], which thus triggers addiction-like eating behaviors [27], including emotional eating, overeating, and loss of control over one’s diet. 

However, to the best of our knowledge, this model has never been tested before. Consequently, robust empirical confirmation is lacking. Indeed, despite the extensive literature, the relationships among CTEs, attachment insecurity, impaired reflective functioning (hypomentalization), and addiction-like eating behaviors remain unclear.

Consequently, on the basis of the abovementioned scientific literature, the aim of this study is to test a multiple mediation model in which CTEs (X) are associated with attachment anxiety (M1a), attachment avoidance (M1b), and hypomentalization (M2). In turn, hypomentalization (M2) should be associated with emotional eating (M3). Finally, emotional eating should be simultaneously associated with addiction-like eating behaviors such as overeating (Y1) and reduced dietary control (Y2). Explicit hypotheses about each path (relationship) between variables were formulated:

**H1**: CTEs, attachment anxiety, attachment avoidance, hypomentalization, emotional eating, overeating, and reduced dietary control are positively correlated with each other.

**H2**: CTEs statistically predict emotional eating via attachment anxiety, attachment avoidance and hypomentalization.

**H3**: CTEs statistically predict overeating behaviors and reduced dietary control via attachment anxiety, attachment avoidance, hypomentalization and emotional eating.

In other words, it was hypothesized that the positive associations between CTEs and addiction-like eating behaviors would be mediated by the presence of insecure attachment, hypomentalizing, and emotional eating.

## Methods and materials

### Procedure

Participants were recruited from the general population—a convenience sample—through snowball sampling [97] via social media platforms such as Facebook and Twitter/X. The inclusion criteria were as follows: (A) aged 18 years or older; (B) native Italian speaker; (C) full completion of the assessment battery (only compete submission were recorded; namely, no missing data); and (D) a total completion time between 10 and 20 min. In particular, this final criterion was established through a pilot study conducted with 20 participants (not included in the final sample) to assess the clarity of the questionnaires and determine an appropriate completion time. This approach aimed to prevent excessively fast responses (which could indicate random or automatic answers, or bots and/or imposters), or overly slow responses (for example, if the participant had been interrupted or was distracted) [98].

Moreover, in line with current recommendations, data quality screening was conducted prior to statistical analyses to ensure the validity of the online responses [98]. In the preliminary instructions for accessing the study, the participants were clearly informed that they would not receive any form of compensation.

All the participants took part voluntarily and all participants provided informed consent before accessing the survey. The study was approved by the Ethics Committee of the University of Padua (protocol no. 547-a) and was conducted in accordance with the institution’s ethical standards.

### Sample size determination

The required sample size was estimated through Monte Carlo simulation, an approach particularly suited for structural equation models involving multiple latent variables and indirect effects [99, 100]. The simulations were conducted in R via the lavaan package [101], following established recommendations for SEM power analysis [102, 103]. For each candidate sample size, ranging from 400 to 1200 in increments of 50, 1000 datasets were generated on the basis of population parameters consistent with theoretical expectations and prior research. These included moderate factor loadings, structural paths and indirect effects of realistic magnitude (small-to-medium, approximately 0.15–0.30) across mediators and outcomes. Power was defined as the proportion of replications in which at least 80% of the key structural coefficients reached significance at *p* < 0.05, a criterion that balances statistical sensitivity with model complexity. To ensure estimation stability, a minimum convergence rate of 90% was also needed. The estimation relies on maximum likelihood (ML), which is quite robust to violations of multivariate normality [104]. On the basis of these criteria, the simulation indicated that a minimum sample size greater than 800 participants would be sufficient for correctly estimating model parameters.

### Participants

The sample comprised 1014 participants. The sample included 138 males (13.6%) and 876 females (86.4%), aged between 18 and 74 years (*mean* = 44.11, *SD* = 13.70), with a BMI ranging from 16.14 to 47.75 kg/m^2^ (*mean* = 24.71 kg/m^2^, *SD* = 4.98). More details are provided in Table 1.

**Table 1:** Sample descriptive statistics

	Descriptives (*N* = 1014)	
Age (*mean*,* SD*)	44.11	13.70
BMI (Kg/m^2^) (*mean*,* SD*)	24.71	4.98
Sex (*n*, %)		
Male	138	13.6%
Female	876	86.4%
Education (*n*, %)		
Middle school	98	9.7%
High school	495	48.8%
Bachelor	345	34.0%
Master degree/PhD	76	7.5%
Work status (*n*, %)		
Student	114	11.2%
Full-time worker	534	52.7%
Entrepreneur	208	20.5%
Unemployed	87	8.6%
Retired	71	7.0%
Civil status (*n*, %)		
Single	164	16.2%
In a relationships	280	27.6%
Married	473	46.6%
Separated/divorced	80	7.9%
Widowed	17	1.7%
BMI class (*n*, %)		
Underweight (16–18.49)	46	4.5%
Normal weight (18.5–24.99)	563	55.5%
Overweight (25–29.99)	266	26.2%
Class I obesity (30–34.99)	89	8.8%
Class II obesity (35–39.99)	40	3.9%
Class III obesity (> 40)	10	1.0%
Eating disorder (*n*, %)		
No ED	912	89.9%
Anorexia nervosa (AN)	23	2.3%
Bulimia nervosa (BN)	12	1.2%
Binge eating disorder (BED)	36	3.6%
Otherwise specified feeding and eating disorder (OSFED)	31	3.1%

## Measures

### Childhood trauma questionnaire–short form (CTQ-SF)

The CTQ-SF [40] is a self-reported measure designed to assess traumatic childhood experiences. It includes 28 items rated on a 5-point Likert scale ranging from 1 (= “*Never true*”) to 5 (= “*Very often true*”). Of these, 25 items are grouped into five subscales that assess different domains of maltreatment, namely, emotional abuse, physical abuse, sexual abuse, emotional neglect, and physical neglect, with each domain represented by five items. The remaining three items function as a minimization/denial scale, capturing the tendency to underreport traumatic events. A total score reflecting overall childhood trauma can be obtained by summing the subscale scores. Higher scores indicate a greater presence of childhood traumatic experiences. In the present study, the Italian version of the CTQ-SF was used [105], which demonstrated excellent internal consistency, with McDonald’s omega = 0.925.

### Relationship questionnaire (RQ)

The RQ [72] is one of the most commonly used self-report measures for evaluating adult attachment styles [70, 71]. Individuals rate how well four brief descriptions reflect their typical relationship style, using a 7-point Likert scale ranging from 1 (= “strongly disagree”) to 7 (= “strongly agree”). Each description corresponds to one of the four prototypical adult attachment styles: secure (positive view of self and others), dismissing (positive view of self, negative view of others), preoccupied (negative view of self, positive view of others), and fearful (negative view of both self and others). In line with the standard scoring procedure [72], the two core dimensions of insecure attachment—anxiety and avoidance—were derived by combining responses to the four prototypes of anxiety [(fearful + preoccupied) - (secure + dismissing)] and avoidance [(fearful + dismissing) - (secure + preoccupied)]. As these dimensions represent computed scores rather than multi-item scales, traditional internal consistency estimates (e.g., McDonald’s omega) are not applicable. Higher scores indicate greater levels of either anxious or avoidant attachment, depending on the dimension assessed. Higher scores indicate greater levels of either anxious or avoidant attachment, depending on the dimension assessed. For the present study, the Italian version of the RQ was used [106].

### Reflective functioning questionnaire (RFQ)

The RFQ [107] is a brief self-report questionnaire designed to assess a specific mentalization referred to as reflective functioning, namely, the capacity to interpret and understand one’s own and others’ mental states [81]. It comprises eight items rated on a 7-point Likert scale ranging from 1 (= “*completely disagree*”) to 7 (= “*completely agree*”), where respondents express their agreement with statements related to mentalizing processes. The questionnaire captures two contrasting dimensions: certainty (i.e., hypermentalizing—excess reflective functioning) and uncertainty (i.e., hypomentalizing—lack of reflective functioning) about mental states. In this study, the uncertainty subscale was used, which reflects impairments in reflective functioning. Higher scores indicate greater difficulty in understanding mental states. The Italian version of the RFQ [108] was used, and it showed good internal consistency, with McDonald’s omega = 0.772.

### Three-factor eating questionnaire-revised 18 (TFEQ-R-18)

The TFEQ-R-18 [109] is a widely used self-report measure designed to evaluate three primary dimensions of eating behavior: cognitive restraint (CR), uncontrolled eating (UE), and emotional eating (EE). The questionnaire comprises 18 items rated on a 4-point Likert scale, with higher scores indicating stronger tendencies in the corresponding domain. The CR subscale assesses individuals’ efforts to restrict food intake to manage body weight or shape. The UE subscale reflects a loss of control over eating, often resulting in episodes of overeating. The EE subscale captures the propensity to eat in response to negative emotional experiences. The present study employed the Italian version of the TFEQ-R-18 [110] and only the EE dimension, which is in line with the study objectives. The EE scale demonstrated good internal consistency: McDonald’s omega = 0.888.

### Addiction-like eating behavior scale (AEBS)

The AEBS [111] is a 15-item self-report instrument developed to assess addictive patterns of eating behavior, namely, food addiction–like behaviors. The items are rated on a 5-point Likert scale ranging from 1 (= “*strongly disagree/never*”) to 5 (= “*strongly agree/always*”). The scale includes two dimensions: appetite drive (AD), which reflects food addiction–related behaviors such as heightened responsiveness to food-related rewards and tendencies toward overeating, and low dietary control (LDC), which captures reduced self-regulation in managing eating impulses. Higher scores on each subscale indicate more pronounced difficulties in the respective area. Additionally, a general score can be computed. The AEBS has shown good psychometric properties in evaluating maladaptive eating patterns. In the present study, the validated Italian version of the AEBS [112] was used, with both dimensions demonstrating excellent internal consistency: McDonald’s omega = 0.879 and McDonald’s omega = 0.857 for AD and LDC, respectively.

### Statistical analysis

Statistical analyses were performed via R software. According to the predefined inclusion criteria, none of the observations provided missing values. Consistent with prior research, a series of preparatory steps were completed before the structural equation model (SEM) was estimated [113, 114]. Moreover, as SEM relies on the general linear model framework, its underlying assumptions were examined and found to be met.

*First*, as preliminary analyses, Pearson correlation coefficients (*r*) were calculated to examine the strength of the relationships among variables and identify potential issues. Correlation coefficients were interpreted via Cohen’s benchmarks [115]: *r* < 0.10, trivial; *r* from 0.10 to 0.30, small; *r* from 0.30 to 0.50, moderate; and *r* > 0.50, large. Correlations exceeding |0.80| were considered indicative of potentially problematic overlaps [116].

*Second*, the factorial structure (i.e., measurement model) of each questionnaire was assessed via confirmatory factor analysis (CFA), with the aim of verifying whether the observed data conformed to the theoretical structure established in previous validation studies. The scales tested included the CTQ-SF, the RFQ uncertainty, and the AEBS. Importantly, the factorial structure was not tested for the RQ scale. Indeed, consistent with the original validation study, a factorial structure for the RQ cannot be meaningfully evaluated. CFAs were performed via the diagonally weighted least squares (DWLS) estimator [117, 118]. Model adequacy was evaluated via fit indices and their thresholds [118–120]: (A) a nonsignificant χ^2^ test (*p* > 0.05) was desirable; (B) RMSEA values below 0.08 indicated acceptable fit; (C) CFI values above 0.90 suggested a satisfactory fit; and (D) SRMR values below 0.08 were interpreted as indicative of good model fit.

*Third*, Harman’s single-factor test was conducted [121] to assess the potential presence of common method bias, namely, whether a single latent factor accounts for the majority of the covariance among observed variables. First, a model with correlated latent factors was specified, reflecting the theoretical structure of the included scales. Next, a constrained model was tested in which all the items were loaded onto a single latent factor. The two models were then compared using established thresholds for changes in fit indices: a non-significant change in χ^2^ (Δχ^2^, *p* > 0.05), along with ΔCFI ≤ 0.01 and ΔRMSEA ≤ 0.015, was used to assess model deterioration [122]. These comparisons followed standard interpretive criteria [122]. A significantly better fit of the correlated-factor model over the single-factor model would indicate that common method bias is unlikely to be a concern.

*Fourth*, latent variables were represented via item parcels as indicators. This approach offers several psychometric benefits, including improved reliability and a reduced likelihood of violating assumptions such as normality [123]. A partially disaggregated parceling strategy was adopted, following established guidelines [124]. Specifically, for the majority of the scales (‘RFQ uncertainty’, ‘AD’, and ‘LDC’), parcels were constructed via the ‘item-to-construct balance’ method [123, 125]. For the CTQ-SF, parcels were created via the ‘domain-representative strategy’ [123, 125]. For each latent construct, a minimum of three parcels was created to ensure that the model was at least just identified [118, 123, 126]. After parcel creation, descriptive statistics were checked to ensure that the parcel scores did not display excessive skewness (Sk >|3|) or kurtosis (K >|10|), in line with recommended thresholds [118]. No item parcels were created for the EE scale, as it consists of only three items. Finally, considering that RQ is composed of two single items/dimensions (i.e., attachment anxiety and attachment avoidance, see dedicated section), two single-item latent variables were created via the reliability correction approach [124, 127, 128], preserving its theoretical meaning. In accordance with the procedure of Jorgensen [129] and following established recommendations [124, 127, 128], the residual variance of each single-item indicator was fixed to a value equal to the items’ total variance multiplied by 1-α (α = item internal consistency). Alpha (α) was established a priori (0.75)^1^ according to established and conventional recommendations for single-item reliability [130–132].

**Fig. 1:**
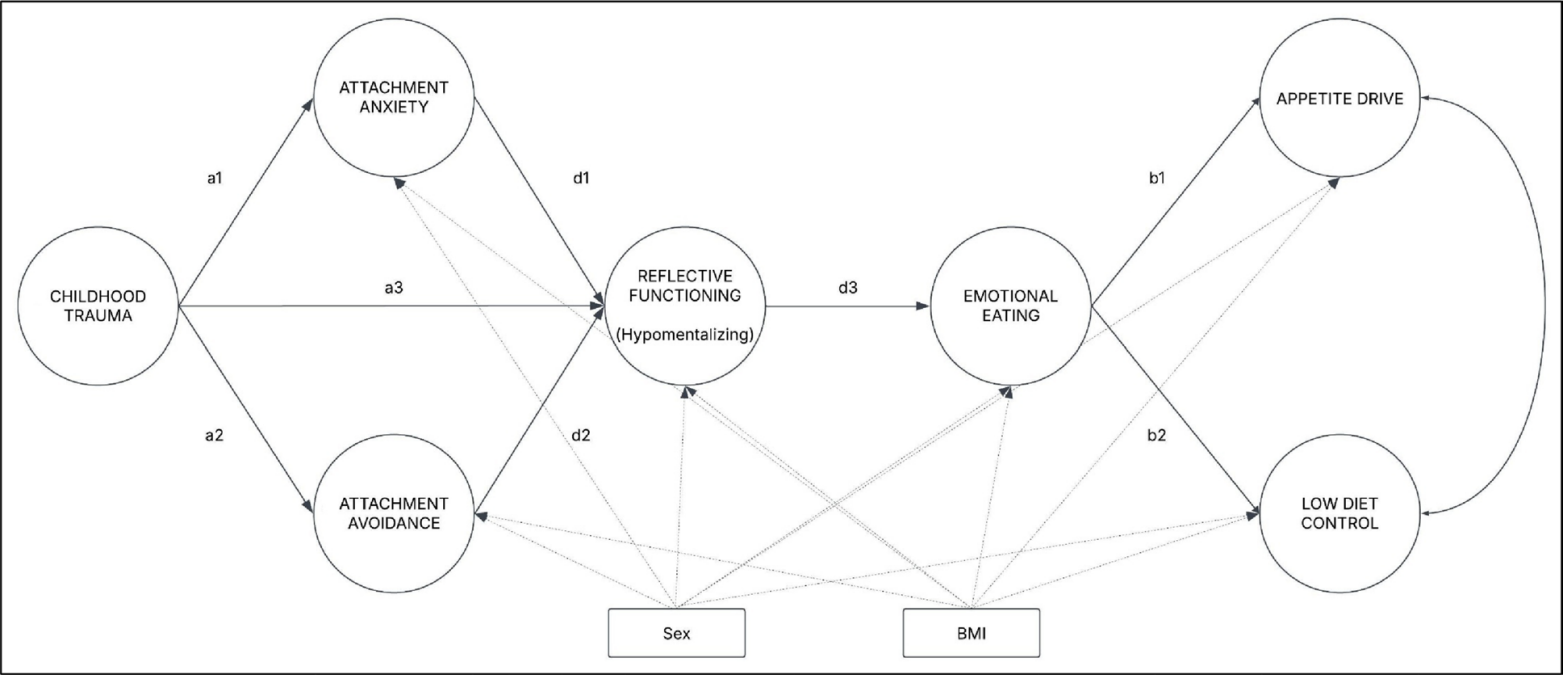
SEM conceptual representation. Sex and BMI were included as covariates. For the sake of clarity in the presentation of this graph, item parcels and indirect effects (e.g., childhood trauma → appetite drive) were not included; only direct effects were depicted. The circles represent latent variables, whereas the rectangles represent observed variables

*Fifth*, an SEM with latent variables was estimated, incorporating a predictor, four mediators, and two outcome variables [126, 133]. Specifically, childhood trauma (X) was modeled as a predictor of addiction-like eating behaviors (Y1) and low diet control (Y2), with attachment anxiety (M1a), attachment avoidance (M1b), reflective functioning (M2) and emotional eating (M3) serving as mediating variables (see Fig. 1). Sex and BMI were used as covariates. The model was estimated via the maximum likelihood (ML) estimator. Given that not all parcel indicators followed a perfectly normal distribution, a nonparametric bootstrapping procedure with 10,000 resamples and Bollen–Stine correction was applied [118, 134, 135]. Model fit was assessed via the standard set of fit indices (χ^2^, RMSEA, CFI, and SRMR) and their conventional thresholds [118, 120]. All regression coefficients (β) presented in the results are unstandardized.

## Results

### Preliminary analysis

Correlation analyses revealed statistically significant linear associations of varying strength—from small to large—among the psychological variables included in the mediation model. Childhood trauma showed moderate positive associations with both attachment anxiety (*r* = 0.315) and hypomentalizing (*r* = 0.241) and a weaker correlation with attachment avoidance (*r* = 0.156). It was also positively associated with all the eating-related variables (i.e., emotional eating, appetite drive, and low diet control), with correlations ranging from *r* = 0.205 to *r* = 0.347. Attachment anxiety was positively correlated with hypomentalizing (*r* = 0.260) and with all eating-related variables (*r* range: 0.188 to 0.245). In contrast, attachment avoidance did not show significant associations with any variables included in the mediation model. Hypomentalizing was significantly associated with all eating-related variables, with correlations ranging from *r* = 0.217 to *r* = 0.302. Moreover, emotional eating, appetite drive, and low diet control showed moderate-to-large intercorrelations, with coefficients ranging from *r* = 0.352 to *r* = 0.610. With respect to BMI, no significant correlations were found with childhood trauma, attachment dimensions, or hypomentalizing, except for a small positive association between BMI and hypomentalizing. Moderate positive associations emerged between BMI and emotional eating, appetite drive, and low perceived diet control. The results are reported in Table 2.

**Table 2:** Correlations among variables

		Descriptives				Correlations								
		M	SD	Sk	K	1	2	3	4	5	6	7	8	9
1	Childhood trauma	37.59	11.53	1.37	2.16	–								
2	Attachment anxiety	− 1.18	4.03	0.27	0.18	0.315^***^	–							
3	Attachment avoidance	0.83	3.65	0.24	0.09	0.156^***^	0.132^***^	–						
4	Hypomentalizing	2.95	3.52	1.44	1.80	0.241^***^	0.260^***^	0.034	–					
5	Emotional eating	6.69	2.71	0.25	− 0.98	0.275^***^	0.245^***^	0.020	0.271^***^	–				
6	AEBS total score	32.38	9.80	0.92	1.02	0.327^***^	0.226^***^	− 0.001	0.302^***^	0.571^***^	–			
7	Appetite drive	16.75	6.19	1.45	2.24	0.347^***^	0.188^***^	0.001	0.297^***^	0.610^***^	0.888^***^	–		
8	Low diet control	15.63	5.16	0.26	− 0.41	0.205^***^	0.204^***^	− 0.003	0.217^***^	0.352^***^	0.834^***^	0.487^***^	–	
9	BMI	24.71	4.98	1.11	1.34	0.052	0.007	− 0.046	0.072^*^	0.346^***^	0.380^***^	0.412^***^	0.227^***^	–

*M* mean,* SD* standard deviation,* Sk* skewness,* K* kurtosis. Childhood Trauma = CTQ-SF Total score; Attachment anxiety = attachment anxiety dimension of the RQ; Attachment avoidance = attachment avoidance dimension of the RQ; Hypomentalizing = uncertainty scale of the RFQ; Emotional Eating = emotional eating scale of the TFEQ-R-18; AEBS Total score = total score of the AEBS; Appetite Drive = AD scale of the AEBS; Low diet control = LDC of the AEBS;* BMI* body mass index

*** *p* < 0.001; * *p* < 0.050

### Measurement models—factorial structure analysis

The CTQ-SF (childhood trauma) showed adequate goodness-of-fit indices: χ^2^ (270) = 935.216; *p* < 0.001; RMSEA = 0.049; 90% CI [0.046; 0.053]; *p*(RMSEA < 0.05) = 0.622, CFI = 0.995, SRMR = 0.073. Additionally, the RFQ uncertainty (hypomentalizing) scale showed adequate goodness-of-fit indices: χ^2^ (9) = 55.532; *p* < 0.001; RMSEA = 0.071; 90% CI [0.054; 0.090]; *p*(RMSEA < 0.05) = 0.022, CFI = 0.985, SRMR = 0.068. Finally, the appetite drive and low-diet control scales (AEBS) showed adequate fit indices: χ^2^ (74) = 316.806; *p* < 0.001; RMSEA = 0.057; 90% CI [0.051; 0.063]; *p*(RMSEA < 0.05) = 0.037; CFI = 0.996; SRMR = 0.047. As noted in the statistical analysis section, evaluating the factorial structure of the RQ is inappropriate. Additionally, the emotional eating (EE) subscale of the TFEQ-R-18 consists of only three items, resulting in a saturated model with perfect fit.

### Harman’s single-factor test

A single-factor test revealed the absence of ‘common method bias’. The model with correlated factors provided adequate fit indices: χ^2^ (1091) = 4394.998; *p* < 0.001; RMSEA = 0.055; 90% CI [0.053; 0.056]; *p*(RMSEA < 0.05) < 0.001, CFI = 0.986, SRMR = 0.066. In contrast, the single-factor model provided poor fit indices: χ^2^ (1127) = 43431.177; *p* < 0.001; RMSEA = 0.192; 90% CI [0.191; 0.194]; *p*(RMSEA < 0.05) < 0.001, CFI = 0.820, SRMR = 0.216. The model comparison further suggested the absence of ‘common method bias’: Δχ^2^ (36) = 39,036, *p* < 0.001; |ΔRMSEA| = 0.138, and |ΔCFI| = 0.166.

### Structural equation model with latent variables and item parcels

All item pairs were statistically significantly associated with their latent variable. As reported in Table 3, their standardized factor loadings range from a minimum of 0.316 to a maximum of 0.901. After confirming that the indicators appropriately captured the underlying latent construct, the SEM was examined. Sex and BMI were included as covariates in the SEM.

**Table 3:** Descriptive statistics and factor loadings (λ) of the item parcels

	Descriptives				Item parcel/latent variable relationship				
	M	SD	Sk	K	λ(se)	z value	*p* value	λ*	*R* ^2^
*Childhood trauma (X)*									
pCTQ#1	1.33	0.43	1.89	4.92	1.000			0.642	0.413
pCTQ#2	1.67	0.81	1.43	1.70	2.476 (0.152)	16.262	< 0.001	0.846	0.716
pCTQ#3	2.21	0.92	0.64	− 0.27	2.748 (0.184)	14.901	< 0.001	0.822	0.676
pCTQ#4	1.17	0.42	3.64	16.85	0.828 (0.084)	9.853	< 0.001	0.548	0.301
pCTQ#5	1.15	0.49	4.46	22.65	0.560 (0.095)	5.911	< 0.001	0.316	0.100
*Attachment anxiety (M1a)*									
Att. Anx.	− 1.18	4.03	0.27	0.18	1.000			0.866	0.750
*Attachment avoidance (M1b)*									
Att. Av.	0.83	3.65	0.24	0.09	1.000			0.866	0.750
*Hypomentalizing (M2)*									
pRFQ#1	0.40	0.65	1.83	3.04	1.000			0.686	0.498
pRFQ#2	0.58	0.79	1.35	1.01	1.243 (0.098)	12.717	< 0.001	0.706	0.597
pRFQ#3	0.49	0.69	1.43	1.55	1.185 (0.087)	13.622	< 0.001	0.772	0.471
*Emotional eating (M3)*									
EE#1	2.42	1.03	0.02	− 1.17	1.000			0.841	0.707
EE#2	2.25	1.02	0.19	− 1.15	1.058 (0.027)	39.272	< 0.001	0.901	0.812
EE#3	2.02	0.96	0.47	− 0.88	0.888 (0.026)	33.701	< 0.001	0.808	0.652
*Appetite drive (Y1)*									
pAD#1	2.40	0.90	0.43	− 0.21	1.000			0.699	0.488
pAD#2	2.12	1.01	0.83	− 0.15	1.246 (0.052)	24.049	< 0.001	0.770	0.592
pAD#3	1.54	0.71	1.83	3.89	0.971 (0.047)	20.649	< 0.001	0.853	0.728
pAD#4	1.54	0.68	1.79	3.52	0.959 (0.042)	22.821	< 0.001	0.887	0.786
*Low diet control (Y2)*									
pLDC#1	2.60	0.88	0.28	− 0.28	1.000			0.823	0.677
pLDC#2	2.71	1.02	0.15	− 0.73	1.263 (0.042)	30.125	< 0.001	0.899	0.809
pLDC#3	2.50	0.96	0.52	− 0.21	1.080 (0.041)	26.517	< 0.001	0.819	0.671

*M* mean,* SD* standard deviation,* Sk* skewness, * K* kurtosis, * λ* unstandardized factor loading,* se* standard error, λ* standardized factor loading,* R2* explained variance,* p(…)* item parcel; childhood trauma = CTQ-SF total score; attachment anxiety = attachment anxiety dimension of the RQ; attachment avoidance = attachment avoidance dimension of the RQ; hypomentalization = RFQ uncertainty scale; emotional eating = TFEQ-R-18 EE scale; appetite drive = AEBS AD scale; low diet control = AEBS LDC

**Fig. 2:**
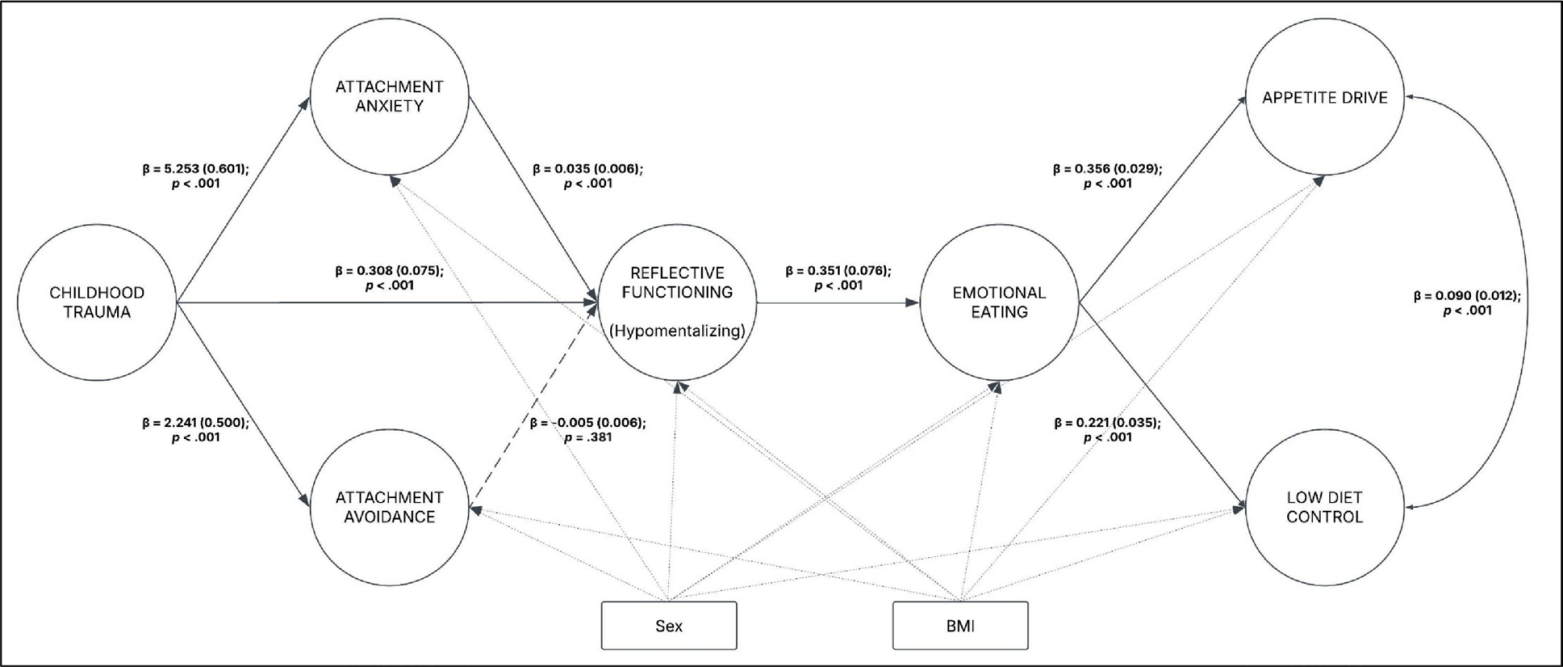
SEM statistical results. Sex and BMI were included as covariates. For the sake of clarity in the presentation of this graph, item parcels and indirect effects (e.g., childhood trauma → appetite drive) were not included; only direct effects were depicted. The circles represent latent variables, whereas the rectangles represent observed variables. *β* = unstandardized beta; SE = standard error; *p* = *p* value

The hypothesized model (Figs. 1 and 2) provided adequate goodness-of-fit indices: χ^2^ (177) = 801.072; *p* < 0.001; RMSEA = 0.059; 90% CI [0.055; 0.063]; *p*(RMSEA < 0.05) < 0.001, CFI = 0.937, SRMR = 0.040. To keep this section concise, only the sequential paths (e.g., X → M1a, M1a → M2, etc.) are reported here. For a comprehensive overview of the results, please refer to Tables 4 and 5.

**Table 4:** Summary of simple regression parameter estimates (β) with 95% confidence intervals (Fig. 2)

	Path	β*	β (SE)	95%CI [L - U]	z value	*p* value	*R* ^2^
Childhood trauma (X) → Attachment Anxiety (M1a)	a1	0.415	5.253 (0.601)	[4.151; 6.523]	8.741	< 0.001	0.175
Childhood trauma (X) → Attachment Avoidance (M1b)	a2	0.195	2.241 (0.500)	[1.302; 3.267]	4.480	< 0.001	0.045
Attachment Anxiety (M1a) → Hypomentalizing (M2)	d1	0.270	0.035 (0.006)	[0.022; 0.048]	5.342	< 0.001	0.158
Attachment Avoidance (M1b) → Hypomentalizing (M2)	d2	− 0.036	− 0.005 (0.006)	[− 0.016; 0.006]	− 0.876	0.381	
Hypomentalizing (M2) → Emotional eating (M3)	d5	0.181	0.351 (0.076)	[0.207; 0.503]	4.599	< 0.001	0.301
Emotional eating (M3) → Appetite drive (Y1)	b1	0.495	0.356 (0.029)	[0.302; 0.414]	12.443	< 0.001	0.535
Emotional eating (M3) → Low diet control (Y2)	b2	0.264	0.221 (0.035)	[0.152; 0.290]	6.333	< 0.001	0.206
Childhood trauma (X) → Appetite drive (Y1)	c1	0.239	0.543 (0.100)	[0.354; 0.745]	5.442	< 0.001	
Childhood trauma (X) → Low diet control (Y2)	c2	0.082	0.217 (0.115)	[− 0.011; 0.439]	1.895	0.058	
Attachment Anxiety (M1a) ↔ Attachment Avoidance (M1b)		0.104	1.021 (0.374)	[0.282; 1.758]	2.730	0.006	
Appetite drive (Y1) ↔ Low diet control (Y2)		0.327	0.090 (0.012)	[0.066; 0.114]	7.399	< 0.001	
Childhood trauma (X) → Hypomentalizing (M2)	a3	0.196	0.318 (0.075)	[0.174; 0.464]	4.259	< 0.001	
Childhood trauma (X) → Emotional eating (M3)	a4	0.183	0.578 (0.131)	[0.332; 0.847]	4.421	< 0.001	
Attachment Anxiety (M1a) → Emotional eating (M3)	d3	0.150	0.037 (0.011)	[0.017; 0.059]	3.475	0.001	
Attachment Anxiety (M1a) → Appetite drive (Y1)	b3	− 0.060	− 0.011 (0.007)	[− 0.024; 0.003]	− 1.564	0.118	
Attachment Anxiety (M1a) → Low diet control (Y2)	b4	0.111	0.023 (0.009)	[0.005; 0.041]	2.456	0.014	
Attachment Avoidance (M1b) → Emotional eating (M3)	d4	− 0.044	− 0.012 (0.010)	[− 0.031; 0.006]	− 1.266	0.205	
Attachment Avoidance (M1b) → Appetite drive (Y1)	b5	− 0.027	− 0.005 (0.006)	[− 0.018; 0.007]	− 0.830	0.407	
Attachment Avoidance (M1b) → Low diet control (Y2)	b6	− 0.042	− 0.010 (0.009)	[− 0.027; 0.008]	− 1.085	0.278	
Hypomentalizing (M2) → Appetite drive (Y1)	b7	0.140	0.196 (0.053)	[0.093; 0.302]	3.684	< 0.001	
Hypomentalizing (M2) → Low diet control (Y2)	b8	0.102	0.165 (0.072)	[0.027; 0.308]	2.310	0.021	
Sex → Attachment Anxiety (M1a)		0.037	0.373 (0.352)	[− 0.323; 1.057]	1.061	0.289	
Sex → Attachment Avoidance (M1b)		0.048	0.445 (0.333)	[− 0.225; 1.092]	1.338	0.181	
Sex → Hypomentalizing (M2)		0.015	0.019 (0.047)	[− 0.077; 0.111]	0.403	0.687	
Sex → Emotional eating (M3)		0.121	0.307 (0.069)	[0.170; 0.442]	4.434	< 0.001	
Sex → Appetite drive (Y1)		− 0.066	− 0.121 (0.041)	[− 0.200; − 0.039]	− 2.937	0.003	
Sex → Low diet control (Y2)		− 0.030	− 0.064 (0.064)	[− 0.192; 0.059]	− 1.007	0.314	
BMI → Attachment Anxiety (M1a)		− 0.009	− 0.006 (0.024)	[− 0.054; 0.040]	− 0.264	0.792	
BMI→ Attachment Avoidance (M1b)		− 0.060	− 0.038 (0.023)	[− 0.083; 0.008]	− 1.615	0.106	
BMI → Hypomentalizing (M2)		0.072	0.006 (0.003)	[− 0.000; 0.013]	1.911	0.056	
BMI → Emotional eating (M3)		0.347	0.060 (0.005)	[0.050; 0.071]	11.365	< 0.001	
BMI → Appetite drive (Y1)		0.186	0.023 (0.004)	[0.015; 0.031]	5.721	< 0.001	
BMI → Low diet control (Y2)		0.121	0.018 (0.005)	[0.008; 0.027]	3.628	< 0.001	

β* standardized beta, * β* unstandardized beta,* SE* standard error,* 95% CI* 95% confidence intervals for the unstandardized beta,* R2* explained variance

**Table 5:** Summary of parameter estimates for the model’s indirect and total effects (Fig. 2)

	Path	β*	β (SE)	95%CI [L - U]	z value	*p* value
Effect of X on Y1 via M1a	a1*b3	− 0.025	− 0.056 (0.037)	[− 0.130; 0.014]	− 1.533	0.125
Effect of X on Y1 via M1b	a2*b5	− 0.005	− 0.012 (0.015)	[− 0.042; 0.017]	− 0.799	0.424
Effect of X on Y1 via M2	a3*b7	0.027	0.062 (0.022)	[0.024; 0.109]	2.885	0.004
Effect of X on Y1 via M3	a4*b1	0.091	0.206 (0.048)	[0.117; 0.304]	4.282	< 0.001
Effect of X on Y1 via M1a and M2	a1*d1*b7	0.016	0.036 (0.013)	[0.014; 0.064]	2.788	0.005
Effect of X on Y1 via M1b and M2	a2*d2*b7	− 0.001	− 0.002 (0.003)	[− 0.009; 0.003]	− 0.757	0.449
Effect of X on Y1 via M1a and M3	a1*d3*b1	0.031	0.070 (0.023)	[0.030; 0.121]	2.996	0.003
Effect of X on Y1 via M1b and M3	a2*d4*b1	− 0.004	− 0.010 (0.008)	[− 0.028; 0.005]	− 1.160	0.246
Effect of X on Y1 via M2 and M3	a3*d5*b1	0.018	0.040 (0.013)	[0.017; 0.068]	3.059	0.002
Effect of X on Y1 via M1a, M2, and M3	a1*d1*d5*b1	0.010	0.023 (0.007)	[0.012; 0.038]	3.374	0.001
Effect of X on Y1 via M1b, M2, and M3	a2*d2*d5*b1	− 0.001	− 0.001 (0.002)	[− 0.005; 0.002]	− 0.810	0.418
Total indirect effect of X on Y1		0.156	0.355 (0.063)	[0.238; 0.486]	5.629	< 0.001
Total effect on Y1		0.396	0.897 (0.100)	[0.711; 1.100]	8.987	< 0.001
Effect of X on Y2 via M1a	a1*b4	0.046	0.121 (0.052)	[0.025; 0.230]	2.308	0.021
Effect of X on Y2 via M1b	a2*b6	− 0.008	− 0.022 (0.021)	[− 0.067; 0.017]	− 1.022	0.307
Effect of X on Y2 via M2	a3*b8	0.020	0.053 (0.026)	[0.008; 0.111]	1.992	0.046
Effect of X on Y2 via M3	a4*b2	0.048	0.128 (0.036)	[0.064; 0.207]	3.527	< 0.001
Effect of X on Y2 via M1a and M2	a1*d1*b8	0.011	0.030 (0.015)	[0.005; 0.062]	2.045	0.041
Effect of X on Y2 via M1b and M2	a2*d2*b8	− 0.001	− 0.002 (0.003)	[− 0.008; 0.002]	− 0.724	0.469
Effect of X on Y2 via M1a and M3	a1*d3*b2	0.016	0.043 (0.015)	[0.018; 0.077]	2.878	0.004
Effect of X on Y2 via M1b and M3	a2*d4*b2	− 0.002	− 0.006 (0.005)	[− 0.018; 0.003]	− 1.116	0.264
Effect of X on Y2 via M2 and M3	a3*d5*2	0.009	0.025 (0.009)	[0.010; 0.044]	2.823	0.005
Effect of X on Y2 via M1a, M2, and M3	a1*d1*d5*b2	0.005	0.014 (0.005)	[0.007; 0.025]	3.076	0.002
Effect of X on Y2 via M1b, M2, and M3	a2*d2*d5*b2	− 0.000	− 0.001 (0.001)	[− 0.003; 0.001]	− 0.800	0.424
Total indirect effect of X on Y2		0.145	0.383 (0.067)	[0.260; 0.525]	5.681	< 0.001
Total effect on Y2		0.228	0.601 (0.103)	[0.401; 0.804]	5.821	< 0.001

β* standardized beta,* β* unstandardized beta,* SE* standard error,* 95% CI* 95% confidence intervals for the unstandardized beta. X = childhood trauma; M1a = attachment anxiety; M1b = attachment avoidance; M2 = hypomentalizing; M3 = emotional eating; Y1 = appetite drive; Y2 = low diet control

Childhood trauma (X) was positively associated with attachment anxiety (M1a), *path a1*: β = 5.253 (SE = 0.601), [95% CI: 4.151; 6.523], z = 8.741, *p* < 0.001. Moreover, childhood trauma (X) was positively associated with attachment avoidance (M1b), *path a2*: β = 2.241 (SE = 0.500), [95% CI: 1.302; 3.267], z = 4.480, *p* < 0.001. Additionally, childhood trauma (X) was positively associated with hypomentalizing (M2), *path a3*: β = 0.318 (SE = 0.075), [95% CI: 0.174; 0.464], z = 4.259, *p* < 0.001.

Attachment anxiety (M1a) was positively associated with hypomentalizing (M2), *path d1*: β = 0.035 (SE = 0.006), [95% CI: 0.022; 0.048], z = 5.342, *p* < 0.001. In contrast, attachment avoidance (M1b) was not positively associated with hypomentalizing (M2), *path d2*: β = − 0.005 (SE = 0.006), [95% CI: − 0.016; 0.006], z = − 0.876, *p* = 0.381. Attachment anxiety (M1a) and attachment avoidance (M1b) were slightly positively associated with each other: β = 1.021 (SE = 0.374), [95% CI: 0.282; 1.758], z = 2.730, *p* = 0.006.

Moreover, hypomentalizing (M2) predicted emotional eating (M3), *path d5*: β = 0.351 (SE = 0.076), [95% CI: 0.207; 0.503], z = 4.599, *p* < 0.001.

Finally, emotional eating (M3) was positively associated with appetite drive (Y1), *path b1*: β = 0.356 (SE = 0.029), [95% CI: 0.302; 0.414], z = 12.443, *p* < 0.001. Moreover, emotional eating (M3) was positively associated with low-level diet control (Y2), *path b2*: β = 0.221 (SE = 0.035), [95% CI: 0.152; 0.290], z = 6.333, *p* < 0.001. The appetite drive (Y1) and low-diet control (Y2) were moderately positively associated with each other: β = 0.090 (SE = 0.012), [95% CI: 0.066; 0.114], z = 7.399, *p* < 0.001.

Furthermore, an examination of the four indirect paths was performed (the detailed results are reported in Table 5). The effects on appetite drive (Y1) were primarily examined. The *first* indirect effect (controlling for M1b and Y2: childhood trauma → attachment anxiety → hypomentalizing → emotional eating → appetite drive) was statistically significant: β = 0.023 (SE = 0.007), [95% CI: 0.012; 0.038], z = 3.374, *p* = 0.001. In contrast, the *second* indirect effect (controlling for M1a and Y2: childhood trauma → attachment avoidance → hypomentalizing → emotional eating → appetite drive) was not statistically significant: β = − 0.001 (SE = 0.002), [95% CI: − 0.005; 0.002], z = − 0.810, *p* = 0.418. The total indirect effect (controlling for Y2: childhood trauma → attachment anxiety → hypomentalizing → emotional eating → appetite drive *plus* attachment avoidance) was statistically significant: β = 0.355 (SE = 0.063), [95% CI: 0.238; 0.486], z = 5.629, *p* < 0.001.

Second, the effects on the low dietary control (Y2) were examined. The first indirect effect (controlling for M1a and Y1: childhood trauma → attachment anxiety → hypomentalizing → emotional eating → low diet control) was statistically significant: β = 0.014 (SE = 0.005), [95% CI: 0.007; 0.025], z = 3.076, *p* = 0.002. In contrast, the second indirect effect (controlling for M1a and Y1: childhood trauma → attachment avoidance → hypomentalizing → emotional eating → low diet control) was not statistically significant: β = − 0.001 (SE = 0.001), [95% CI: − 0.003; 0.001], z = − 0.800, *p* = 0.424. The total indirect effect (controlling for Y1: childhood trauma → attachment anxiety → hypomentalizing → emotional eating → appetite drive plus attachment avoidance) was statistically significant: β = 0.383 (SE = 0.067), [95% CI: 0.260; 0.525], z = 5.681, *p* < 0.001.

The degree of explained variance (*R*^2^) was 53.5% (*R*^2^ = 0.535) for appetite drive (Y1) and 20.6% (*R*^2^ = 0.206) for low-diet control (Y2).

## Discussion

The present study sought to address a critical gap in the literature by proposing and testing a theory-driven multiple mediation model that links CTEs to addiction-like eating behaviors (i.e., overeating/appetite drive and low diet control) through impairments in reflective functioning (i.e., hypomentalization) and the use of maladaptive self-regulation strategies, such as emotional eating.

Previous research has identified the dual transdiagnostic nature of food addiction and addiction-like eating behaviors [136–138]. Early psychological vulnerabilities such as CTEs, attachment insecurity, and impaired reflective functioning have also been recognized as risk factors for disordered eating [27, 29, 49].

However, no comprehensive model has integrated these variables to explain the developmental pathway from early adversity to addiction-like eating behaviors [27, 91]. Understanding these mechanisms within a trauma-informed framework is essential for clarifying how early relational trauma and deficits in reflective functioning shape disordered eating patterns [49, 84].

The findings of the present study fully support the hypothesized multiple mediation model and are consistent with previous literature emphasizing the key role of early traumatic experiences in the development of disordered and addiction-like eating behaviors [27, 49] as well as reduced diet control. Empirical support was provided for the proposed developmental model [29, 30, 41], in which the effects of CTEs on addiction-like eating behaviors and low diet control were mediated by attachment anxiety, hypomentalization, and emotional eating. Statistically significant associations were observed in the expected directions among all study variables, suggesting that this pathway is shaped by deficits in both interpersonal functioning and self-regulation [27, 49, 81].

The main findings of this study revealed several key points that substantially advance the understanding of trauma-based pathways to addiction-like eating behaviors and low diet control. First, in line with previous studies [49], CTEs demonstrated significant positive associations with both attachment anxiety and hypomentalizing, which is consistent with attachment theory’s predictions regarding the impact of early relational trauma on internal working models and mentalizing capacity [42, 66, 69]. Notably, while CTEs predict both dimensions of attachment insecurity, only attachment anxiety—not attachment avoidance—has emerged as a significant predictor of hypomentalizing [49]. This differential pattern aligns with recent theoretical developments suggesting that attachment anxiety, characterized by negative self-views and a heightened need for proximity, may be more directly linked to mentalization difficulties than attachment avoidance, which involves defensive deactivation of the attachment system [49, 70, 71, 73, 74].

The finding that attachment anxiety, but not attachment avoidance, mediates the relationship between CTEs and hypomentalizing has important theoretical implications and is in line with the findings of previous studies [49]. Indeed, individuals with high attachment avoidance may maintain some mentalizing capacity through emotional suppression and cognitive distancing, although this may come at the cost of emotional authenticity and interpersonal connection [74, 139, 140]. In contrast, individuals with high attachment anxiety tend to exhibit hypervigilance to attachment-related threats and intense emotional responses that can overwhelm their mentalizing capacity, particularly during stress [78–81]. This emotional flooding interferes with the individual’s ability to accurately understand both their own and others’ mental states, resulting in rigid and distorted interpretations of internal experiences [78–81, 88], which could lead to impulsive/instinctual and unregulated behaviors.

Moreover, the central role of hypomentalizing (namely, impaired reflective functioning) in the model represents perhaps one of the most significant theoretical contributions of this study. These results align with recent findings [27, 91] that impaired reflective functioning serves as a crucial bridge—via attachment anxiety—between early relational trauma and disordered eating behaviors [49, 59, 84, 86, 141] related to food addictive behaviors (e.g., emotional eating and appetite drive) and low dietary control [27]. When individuals cannot adequately mentalize—that is, when they cannot understand their own and others’ behaviors in terms of underlying mental states—they become vulnerable to using external regulators such as food to manage overwhelming internal states [76, 81, 84]. This finding is consistent with mentalization-based theories of psychopathology, which propose that deficits in reflective functioning (i.e., hypomentalization) create vulnerability to various forms of behavioral dysregulation, including substance use and disordered eating [29, 49, 84].

The pathway from hypomentalizing to emotional eating represents a particularly important mechanism identified in this study, which aligns with recent literature [91]. Emotional eating—the tendency to consume food in response to negative emotional states rather than physiological hunger—emerged as a direct consequence of impaired reflective functioning [92, 93]. When individuals cannot adequately understand and regulate their emotional experiences through mentalizing, they may resort to food as an external regulator to achieve emotional homeostasis [49, 84, 86]. This process is consistent with contemporary literature on FA, which emphasizes the role of UPFs in providing rapid but temporary relief from negative affect, thereby reinforcing cyclical patterns of consumption [8, 13, 16, 17]. The mediating role of emotional eating identified in this study provides important insights into the overlap between addiction-like eating behaviors and other EDs [23, 137] via disordered eating behaviors [8, 27, 31, 32], reinforcing the transdiagnostic nature of emotional eating behaviors [23]. Emotional eating represents a common feature across multiple eating disorder diagnoses and has been identified as a key maintaining factor in both clinical and subclinical eating pathology [93, 94, 96]. The finding that emotional eating—predicted by hypomentalization—serves as the proximal predictor of addiction-like eating behaviors suggests that interventions targeting emotion regulation, mentalizing abilities, and the relationship between affect and eating may be particularly beneficial for individuals with a history of trauma [31, 58–60, 94, 95].

The final links in the proposed model—from emotional eating to both appetite drive and low dietary control—demonstrate how trauma-related vulnerabilities could ultimately manifest as addiction-like eating behaviors related to increased appetite and overeating as well as deficits in dietary self-regulation [23, 24, 34]. In this context, the addiction-like eating behaviors measured in this study—encompassing both heightened overeating/appetite drive and impaired dietary control—reflect the complex profile characteristic of FA. This pattern aligns with the DSM-5 criteria for substance use disorders, which include both approach behaviors (craving, seeking) and control deficits (inability to limit use, continued use despite consequences) [25].

The findings of this study contribute significantly to ongoing debates regarding the conceptualization of FA and its eating-related manifestations [23, 31, 32, 142]. The results suggest that FA shares etiological pathways with both SRADs, EDs, and disordered eating behaviors [33, 35, 37], reinforcing its transdiagnostic nature [23, 25]. The identification of CTEs as a common risk factor, mediated through attachment and difficulties in reflective functioning (i.e., hypomentalization), provides support for trauma-informed approaches to understanding FA, EDs and disordered eating behaviors [41, 43, 61]. Furthermore, as expected, consistent with the correlation analyses, positive associations were observed between BMI and eating-related variables (emotional eating, appetite drive, and low dietary control)—supporting the bidirectional relationship between weight status and eating behavior dysregulation [111, 143]. These findings align with existing literature demonstrating that BMI (model covariate) is linked to difficulties in appetite regulation and dietary self-control [91].

Considering the model as a whole, it is important to emphasize that there is a difference in the predictive capacity of the two outcomes. The difference in explained variance between model outcomes (53.5% for appetite drive versus 20.6% for low dietary control) indicates that the hypothesized model accounts well for eating-related addiction-like behaviors, but other variables (e.g., impulsivity, cognitive restraint, habitual eating routines, sleep quality) may be needed to more comprehensively explain dietary control.

The stronger prediction of appetite drive than of low dietary control suggests that the pathway from trauma to attachment anxiety, hypomentalization and emotional eating may be particularly relevant for understanding the motivational and behavioral aspects of addiction-like eating behavior [41, 144]. Thus, individuals with a history of CTEs may be more likely to develop an attachment anxiety pattern characterized by heightened hypervigilance and preoccupation with external threats [78–81]. This, in turn, may result in the experience and expression of intense and overwhelming emotions—often negative—that impair the self-regulatory functioning of mentalization [30]. As a consequence, the inability to self-regulate in the presence of such emotional states may lead to reliance on an external regulator, such as food [27, 30, 49, 86]. Over time, this regulatory sequence may become rigid and automatized, progressively applied across various stress-inducing contexts regardless of their specific characteristics. As such, food intake may become the individual’s dominant coping strategy in response to the inability to self-regulate [145]. Additionally, the repetitive and compulsive nature of this mechanism—while initially effective in producing short-term relief—may lead to the development of tolerance, requiring progressively larger quantities of food to achieve the same regulatory effect [1, 8, 27, 32]. This process may ultimately contribute to the emergence of addiction-like eating patterns, particularly those related to food intake, while having only a marginal impact on low dietary control, suggesting that additional, and potentially more salient, factors may underlie difficulties in maintaining dietary control.

### Clinical implications

The results of this study have substantial implications for clinical assessment, case formulation, and treatment planning for individuals presenting with addiction-like eating behaviors [27, 49]. The identification of a sequential developmental pathway from CTEs through attachment insecurity and impaired mentalization to emotional eating and addiction-like behaviors suggests several important clinical considerations.

First, a comprehensive assessment of individuals with suspected FA and addiction-like behaviors should include careful evaluation of childhood trauma history, attachment patterns, and mentalizing capacity [49, 63, 86]. These findings suggest that individuals with significant CTEs and attachment anxiety may be at particular risk for developing hypomentalizing and subsequent addiction-like eating behaviors. Clinicians should be aware that problematic eating behaviors may represent attempts at emotional regulation in individuals with trauma histories and mentalization difficulties [27, 49, 56, 84].

Second, the mediation pathway identified in this study points toward specific therapeutic targets [89, 146]. Rather than focusing solely on eating behaviors, treatment approaches should address the underlying psychological vulnerabilities that contribute to addiction-like eating patterns [30, 146–149]. Mentalization-based treatment (MBT) has emerged as a particularly promising intervention, given its focus on enhancing reflective functioning and improving emotional regulation capacity [147]. MBT is a structured intervention that aims to enhance the individual’s capacity to understand behavior in terms of underlying mental states It employs a curious, exploratory therapeutic stance to help individuals recognize and label thoughts, feelings, and desires in themselves and others. Key components include monitoring and regulating emotional arousal to maintain optimal conditions for reflective functioning and foster more secure attachment dynamics [146, 147]. MBT has demonstrated effectiveness for various trauma-related conditions and could be adapted for individuals with FA by incorporating specific attention to eating-related triggers and behaviors [145, 150].

The role of attachment anxiety in the developmental pathway also suggests that therapeutic interventions should address interpersonal difficulties and relationship patterns that may maintain emotional dysregulation and reliance on food for self-soothing [66, 145, 150]. Approaches that combine mentalization-based techniques with attachment-focused interventions may be particularly beneficial for individuals with trauma histories and addiction-like eating behaviors [27].

Additionally, the central role of emotional eating in linking mentalization deficits to addiction-like behaviors suggests that interventions targeting the relationship between emotions and eating may be crucial [151–153]. Dialectical behavior therapy (DBT) skills training, mindfulness-based interventions, and emotion regulation strategies may complement mentalization-focused work by providing concrete tools for managing emotional distress without resorting to food consumption [1, 32, 153].

### Strengths and limitations

However, several limitations should be acknowledged. First, the cross-sectional design precludes causal inferences, and while the theoretical model suggests temporal ordering of variables, longitudinal research is necessary to establish causality definitively. However, in this regard, certain variables, such as CTEs, attachment, and hypomentalization, are inherently unchangeable [56, 154], and as such, their role in statistical models is typically limited to that of predictors. Second, the reliance on self-report measures introduces potential biases, including social desirability response and retrospective recall bias, particularly for CTEs. Moreover, while the CTQ-SF provides a valid assessment of CTEs within the caregiving context, it does not capture the full range of adversities measured by broader ACE questionnaires (e.g., parental divorce, household substance abuse, witnessing domestic violence). Future research should employ more comprehensive assessments of childhood adversity to examine how different types of early life stress contribute to addiction-like eating behaviors. Third, the sample composition, while large, was predominantly female (86.4%)—a common pattern in online survey research using convenience sampling methods—which may limit its generalizability to male populations [155, 156]. Sex differences in trauma responses, attachment patterns, and eating behaviors are well documented, and future research should examine whether the identified pathways operate similarly across sexes. However, the present study sought to address this limitation by including sex as a covariate in the model. Fourth, the convenience sample recruited through social media may not be representative of the broader population, potentially limiting generalizability; however, to mitigate this issue, important checks were carried out to ensure data quality [98]. Fifth, the study focused on a specific set of mediating variables (attachment dimensions, mentalization, emotional eating), whereas other potentially important factors (such as impulsivity, emotion dysregulation, social support, or genetic factors) were not included. Future research should examine how additional variables might modify or enhance the proposed pathway.

Despite these limitations, this study presents several notable strengths that enhance confidence in the findings and their clinical applicability. First, the use of SEM with latent variables and bootstrapped confidence intervals provides robust statistical testing of the hypothesized mediational pathways while accounting for measurement error [118]. Second, the use of item parceling to create latent variables helps address some of the limitations associated with self-report measures by reducing the impact of measurement error and improving model fit [125]. Third, the large sample size (*N* = 1014) provides adequate statistical power for detecting the hypothesized effects and testing complex mediational models [99]. Finally, this study is grounded in well-established theoretical frameworks from attachment theory, mentalization theory, and addiction research, providing strong conceptual foundations for the tested model.

### Future research directions

The findings of this study suggest several important directions for future research [27, 49]. Longitudinal investigations following individuals from childhood through adulthood provide more definitive evidence regarding the temporal sequencing of the proposed pathway and could identify critical periods for intervention. Such studies could also examine how the pathway might differ across developmental stages and identify factors that promote resilience in individuals with trauma histories [49, 92, 147].

Research examining other potential mediators and/or moderators of the identified pathway would enhance the understanding of individual differences in vulnerability and resilience [30, 41]. Factors such as social support, therapeutic intervention, positive relationship experiences, or genetic variations in stress response systems might influence the strength of the associations between trauma, attachment, mentalization, and eating behaviors.

Despite controlling for sex as a covariate, the gender imbalance in the current sample (86.4% female) limits generalizability to males. In the present study, sex showed significant effects on emotional eating and appetite drive but did not significantly affect the mediational pathway from childhood trauma through attachment and mentalization to eating outcomes. Nevertheless, future research should specifically examine sex differences in the trauma-to-addiction pathway [49], as males and females may exhibit different patterns of attachment insecurity, mentalization difficulties, and eating-related coping strategies following childhood trauma [49, 73], with potential implications for targeted interventions.

Additionally, future research should examine the role of emotional eating in response to positive emotions. While the present study focused on emotional eating driven by negative affect—consistent with our trauma-based theoretical framework and the TFEQ-R-18 measurement approach—emotional eating can also occur in response to positive emotions (e.g., celebratory eating, reward-based consumption). Employing measures that distinguish between negative and positive affect-driven eating would allow researchers to examine whether these pathways operate similarly or through different mechanisms, providing a more comprehensive understanding of emotional eating processes in the context of addiction-like eating behaviors.

Clinical research examining the effectiveness of mentalization-based and attachment-focused interventions for individuals with FA would provide important evidence regarding the clinical utility of the theoretical model [27, 145, 146, 153]. Such research could examine whether improvements in mentalization capacity lead to reductions in addiction-like eating behaviors and whether attachment security can be enhanced through therapeutic intervention.

## Conclusions

This study provides the first comprehensive empirical test of a trauma-based developmental model of addiction-like eating behaviors, offering important insights into the complex pathways linking childhood adversity to addiction-like eating behaviors [1, 27]. The findings suggest that the relationship between CTEs and addiction-like eating behaviors operates through a sophisticated network of variables, highlighting the importance of attachment security, mentalization capacity, and emotional eating in understanding eating-related psychopathology.

The identification of hypomentalizing as a central mediator in the pathway from trauma to addiction-like eating behaviors has significant theoretical and clinical implications [16, 76, 146, 157]. These findings support the growing recognition that food addiction and eating disorders share common etiological factors and suggest that trauma-informed, mentalization-based interventions may be particularly beneficial for individuals with addiction-like eating patterns.

The results contribute to the evolving understanding of FA as a legitimate clinical phenomenon that bridges traditional boundaries between substance use disorders and eating disorders [1, 22, 33, 36]. By demonstrating shared developmental pathways with other trauma-related conditions, this research supports integrative approaches to conceptualizing and treating addiction-like eating behaviors [27, 30, 41].

Ultimately, this investigation advances both theoretical understanding and clinical practice by providing empirical support for a comprehensive developmental model of food addiction. The findings underscore the importance of addressing underlying trauma and attachment vulnerabilities in treating addiction-like eating behaviors and suggest that effective interventions should target mentalization capacity and emotional regulation skills rather than focusing solely on eating behaviors or weight management. As the field continues to evolve in its understanding of food addiction and its relationship with eating disorders, this research provides a foundation for more sophisticated, trauma-informed approaches to prevention, assessment, and treatment.

## Data Availability

The datasets presented in this article are not readily available because due to privacy restrictions, data were available from the corresponding author on a reasonable request.

## Footnotes

1 Different SEMs were tested, systematically varying the assumed alpha coefficient from 1 (i.e., assuming no measurement error) to 0.60. The results did not show substantial changes across the models.
